# Graphene Oxide Wrapped Amorphous Copper Vanadium Oxide with Enhanced Capacitive Behavior for High‐Rate and Long‐Life Lithium‐Ion Battery Anodes

**DOI:** 10.1002/advs.201500154

**Published:** 2015-08-07

**Authors:** Kangning Zhao, Fengning Liu, Chaojiang Niu, Wangwang Xu, Yifan Dong, Lei Zhang, Shaomei Xie, Mengyu Yan, Qiulong Wei, Dongyuan Zhao, Liqiang Mai

**Affiliations:** ^1^ State Key Laboratory of Advanced Technology for Materials Synthesis and ProcessingWuhan University of TechnologyWuhan430070China; ^2^ Department of Mechanical and Industrial EngineeringLouisiana State UniversityBaton RougeLA70830USA

**Keywords:** amorphous, anode, copper extraction, copper vanadium oxide, lithium‐ion battery

## Abstract

**Graphene oxide‐wrapped amorphous copper vanadium oxide** is fabricated through a template‐engaged redox reaction followed by vacuum dehydration. This material exhibits high reversible capacity, excellent rate capability, and out standing high‐rate cyclability. The outstanding performance is attributed to the fast capacitive charge storage and the in situ formed copper with enhanced electrical conductivity.

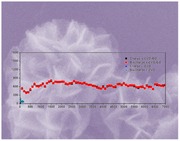

The ever‐increasing consumer market has stimulated the expansion of lithium‐ion batteries from energy storage devices to up‐coming electric vehicles.^1^, ^2^, ^3^, ^4^ To build a better battery, several factors should be taken into account, including energy density, rate capability, cost, safety, and sustainability.^5^, ^6^, ^7^, ^8^, ^9^, ^10^ To achieve this goal, there has been an intensive search interest on new cathode and anode materials with promising electrochemical performance.^11^, ^12^, ^13^, ^14^, ^15^ In the case of anode materials, concerns about the long‐term availability of a few elements have naturally driven researchers toward iron, copper, vanadium, and titanium.^16^, ^17^, ^18^, ^19^, ^20^, ^21^ Moreover, the facile and energy‐saving synthesis, multiple valence states, and rich structural chemistry of vandadium‐based materials have stimulated a great effort toward the development of vanadium‐based electrode materials, including VO_2_,^22^, ^23^, ^24^ V_3_O_7_,^25^ V_6_O_13_,^26^, ^27^ V_2_O_5,_ 28, 29, 30 and metal vanadates.^31^, ^32^

Vanadium belongs to the early transition metal elements. It is generally accepted that vanadium‐based oxides react with lithium through the insertion reaction,^33^, ^34^ while other oxides, such as Fe_2_O_3_ 35 and CuO^36^, ^37^ react with lithium through the conversion reaction. The vanadium‐based oxides have a relatively strong vanadium‐oxygen bond.^33^, ^34^ Thus, the lithiation process can proceed through the insertion reaction without bond cleavage. As for vanadium‐based oxide in its crystalline form, the lithium ions (Li^+^) are able to insert only into the crystallographically defined sites. It has been reported that amorphous MoO_2_ and vanadium oxides with structural defects are able to deliver very stable cycling performance.^33^, ^34^, ^38^ Thus, it is expected that designing amorphous metal vanadates may provide enhanced lithium storage performance as well.

Compared with binary metal oxides, ternary metal oxides have been intensively studied due to the increased electronic conductivity owing to the possible hopping processes and/or defect effect mechanisms.^39^ Kirshenbaum et al.^40^ recently proposed the design of active cathode materials with in situ formed conductive networks. This proposal can reduce or potentially eliminate the need for conductive additives, which do not contribute to the capacity of the cell. Thus, in order to achieve high energy density and good conductivity simutaneously, the rational design of multifunctional ternary metal oxides, in which vanadium acts as the primary redox active center providing multielectron redox reaction and copper as the secondary redox active center during reduction for enhanced elctrical conductivity, is another insightful route.

Herein, we fabricate an amorphous copper vanadaium oxide decorated with graphene oxide (denoted as a‐CVO‐GO) through a facile and environmental‐friendly template‐engaged redox reaction followed by heat treatment in vacuum (**Figure** **1**). The resultant a‐CVO‐GO shows outstanding lithium storage properties in terms of high capacity, superior rate capability, and ultralong cycle life. The a‐CVO‐GO delivers a capacity of 1144 mA h g^−1^ after 100 cycles at 100 mA g^−1^. Even at a high current density of 20 A g^−1^, a capacity of 672.5 mA h g^−1^ is retained after 7000 cycles, suggesting the excellent high‐rate capability and cyclability.

**Figure 1:**
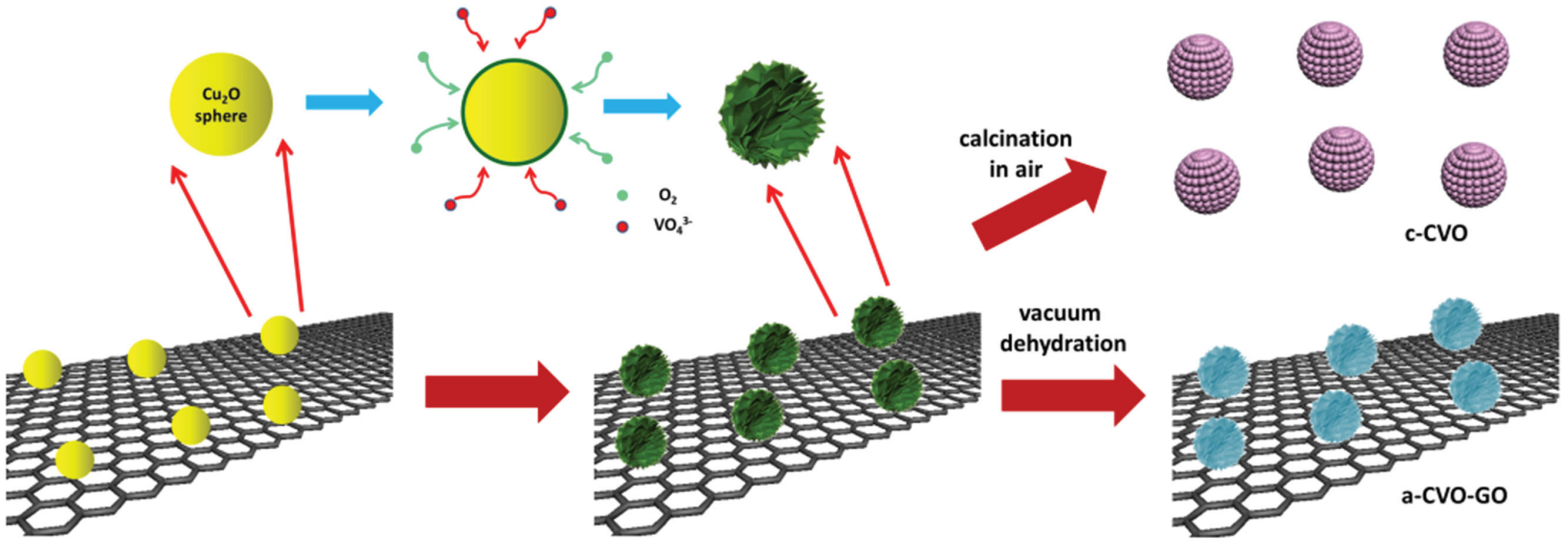
Schematic illustration of the synthesis procedure of a‐CVO‐GO and c‐CVO.

A typical synthesis procedure (Figure 1) starts from the presynthesis of Cu_2_O spheres. The as‐prepared Cu_2_O template is composed of monodispersed nanospheres with a diameter of >>300 nm (Figure S1, Supporting Information). The mixing of NH_4_VO_3_ with Cu_2_O colloidal solution at room temperature leads to the formation of hierarchical Cu_3_V_2_O_7_(OH)_2_·H_2_O nanoflowers with sheet‐like building blocks (Figure S2, Supporting Information). During this process, the Cu_2_O nanospheres act as both the template and the Cu precursor; O_2_ is also involved in the reaction (6 Cu_2_O + 8 VO_3_^−^ + 3 O_2_ + 8 H_2_O = 4 Cu_3_V_2_O_7_(OH)_2_ + 8 OH^−^). The reaction begins at the interfacial region between the Cu_2_O sphere and NH_4_VO_3_ solution. With the inward diffusion of VO_3_^−^ and O_2_, the reaction propagates gradually from the outer surface to the inner core (Figure S3, Supporting Information). Cu_3_V_2_O_7_(OH)_2_ hierarchical nanoflowers can be formed within 10 h.

The amorphous copper vanadium oxide nanoflowers can be obtained by vacuum dehydration of the Cu_3_V_2_O_7_(OH)_2_. H_2_O nanoflowers at 250 °C. X‐ray diffraction (XRD) pattern of the a‐CVO‐GO shows no obvious peaks, indicating the poor‐crystalline or amorphous nature (**Figure** **2**A). To indentify the elemental ratio, the inductively coupled plasma (ICP) is conducted, showing that the Cu/V ratio is about 3:2 (Table S1, Supporting Information). A panoramic view (Figure 2B) and the magnified view (Figure 2C) in the scanning electron microscopic (SEM) images show that the a‐CVO is composed of uniform nanosheet‐constructed nanoflowers with a diameter of around 450 nm, whose morphology is indentical to the Cu_3_V_2_O_7_(OH)_2_. H_2_O precursor. Moreover, the EDX measurements prove that the a‐CVO is composed of Cu, V and O and these three element distributed homogeneously in the selected area (Figures S4 and S5, Supporting Information). The thickness of the nanosheets is estimated to be around 20 nm. The hierarchical nanoflower morphology is further confirmed by transmission electron microscopic (TEM) images (Figure 2D). The magnified TEM image (Figure 2E) shows that the a‐CVO nanosheet is highly porous, which is different from that of the precursor (Figure S2D, Supporting Information). The pore diameter is measured to be around 30–40 nm. The formation of mesopores might be attributed to the dehydration process during which the crystallized water is removed and the sheet is broken down.^23^ No obvious diffraction rings or spots are observed in the selected area electron diffraction (SAED) pattern, indicating the amorphous nature (Figure S6, Supporting Information). To increase the conductivity and structural stability of a‐CVO, graphene oxide (GO) is introduced in the synthesis. The ultrathin and wrinkled graphene oxide sheets wrap on the surface of a‐CVO homogeneously (Figure 2F). This unique a‐CVO‐GO composite structure leads to a surface area of 45.9 m^2^ g^−1^ (Figure S9, Supporting Information). The thermogravimetry (TG) curve reveals that the content of graphene oxide in the a‐CVO‐GO is 8.06 wt% (Figure S7, Supporting Information).

**Figure 2:**
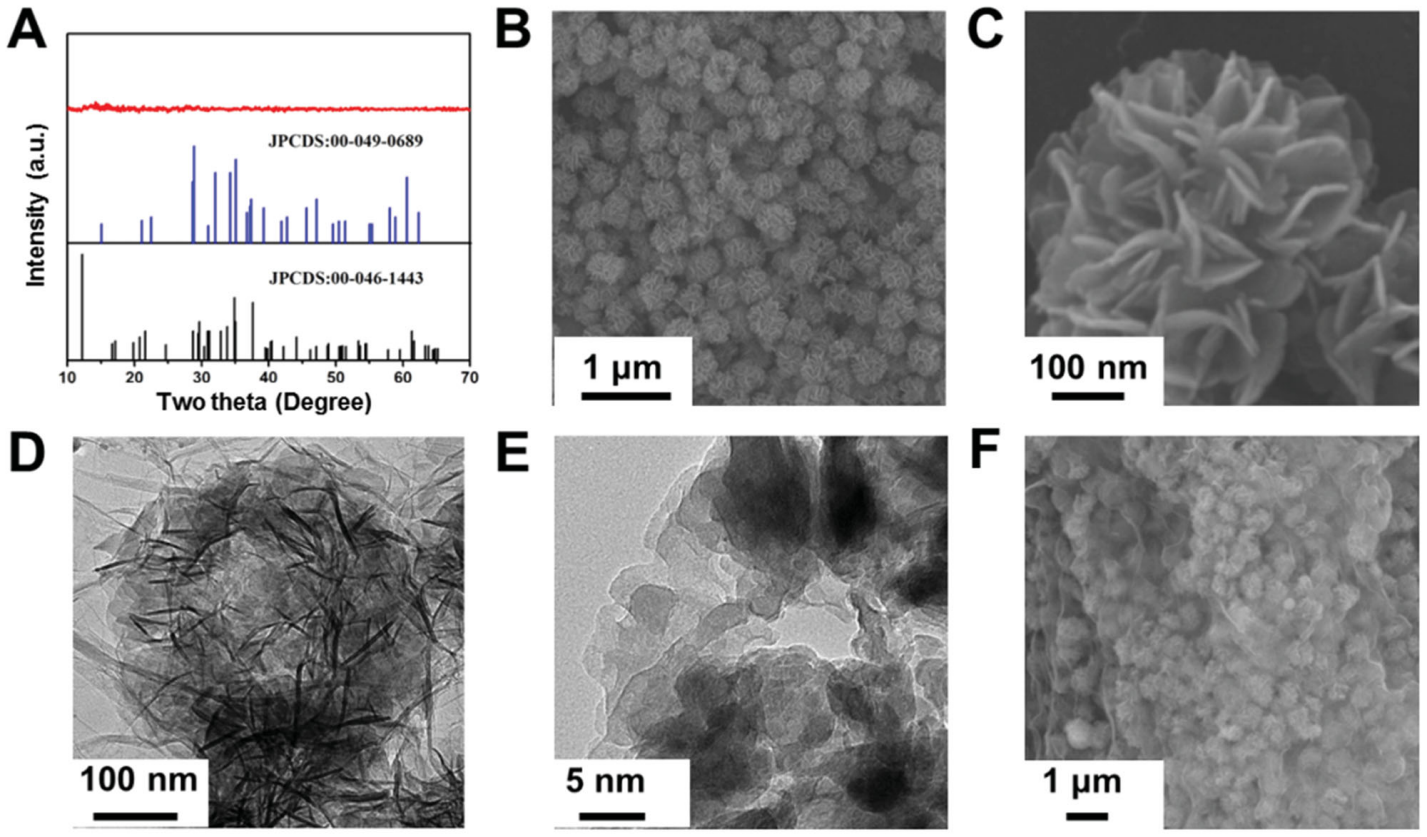
A) XRD pattern of the a‐CVO‐GO. B,C) SEM image and D,E) TEM images of amorphous copper vanadium oxide. F) SEM image of a‐CVO‐GO.

For the purpose of comparison, crystalline copper vanadium oxide (denoted as c‐CVO) is prepared by annealing the precursor in air at 450 °C. The XRD pattern (Figure S8A, Supporting Information) of the c‐CVO matches well with that of Cu_3_V_2_O_8_ (JCPDS card no. 00–049–0689). The c‐CVO (Cu_3_V_2_O_8_) shares the same Cu/V ratio with a‐CVO‐GO (Table S1, Supporting Information). The morphology of c‐CVO is characterized by SEM (Figure S8B, Supporting Information), which shows nanoparticle‐constructed spheres instead of nanosheet‐constructed nanoflowers. No graphene oxide nanosheet is observed on the surface, which indicates that the graphene oxide is removed during the calcination in air. In addition, due to the calcination induced crystallization, the c‐CVO shows a significantly reduced surface area than that of a‐CVO‐GO (15.6 vs 45.9 m^2^ g^−1^, Figure S9, Supporting Information).

The electrochemical performance of a‐CVO‐GO is investigated by assembling CR2016 coin cell using lithium foil as the anode. **Figure** **3**A displays the initial three CV curves at a scan rate of 0.1 mV s^−1^. Generally speaking, the CV curves in the initial three cycles almost overlap, indicating the excellent reversibility. The charge–discharge curves at a current density of 100 mA g^−1^ are presented in Figure 3B. The initial discharge and charge capacities are 830 and 912 mA h g^−1^, respectively. The initial columbic efficiency reaches 109%, which is much higher than that of other transition metal oxide anodes based on conversion reaction mechanism. Similar anomalously high columbic efficiency has been observed in graphite anode by Dahn et al.^41^ The high initial columbic efficiency indicates that the a‐CVO‐GO is a very promising anode material for lithium ion battery. Additionally, the charge–discharge curves of a‐CVO‐GO show several slopes without any obvious plateaus, which may be associated with the discontinuous phase transitions of the host material upon Li^+^ insertion.^42^ At a low current density of 100 mA g^−1^ (Figure 3C), the initial capacity reaches 914 mA h g^−1^. The capacity increases gradually with cycling and reaches 1145 mA h g^−1^ after 100 cycles with no sign of capacity decay, demonstrating the excellent cyclability. The increased capacity might be attributed to the following reason: during discharge/charge cycles, the active material becomes smaller due to electrochemical milling effect^43^ and more active sites are exposed for lithium intercalation/deintercalation (Figure S10, Supporting Information). Further, the rate performance (Figure 3D) reflects that the a‐CVO‐GO exhibits capacities of 757, 672, 659, 598, and 561 mA h g^−1^ at current densities of 0.3, 0.8, 1, 2, and 3 A g^−1^, respectively. When the current density is recovered from 3 to 0.3 and 1 A g^−1^ capacities of 807 and 768 mA h g^−1^ are achieved, respectively. Compared to c‐CVO, the rate capability of a‐CVO‐GO has been significantly improved. Additionally, the capacities of a‐CVO‐GO at each current density all witness a small increase, which is in agreement with the cycling performance at 100 mA g^−1^.

**Figure 3:**
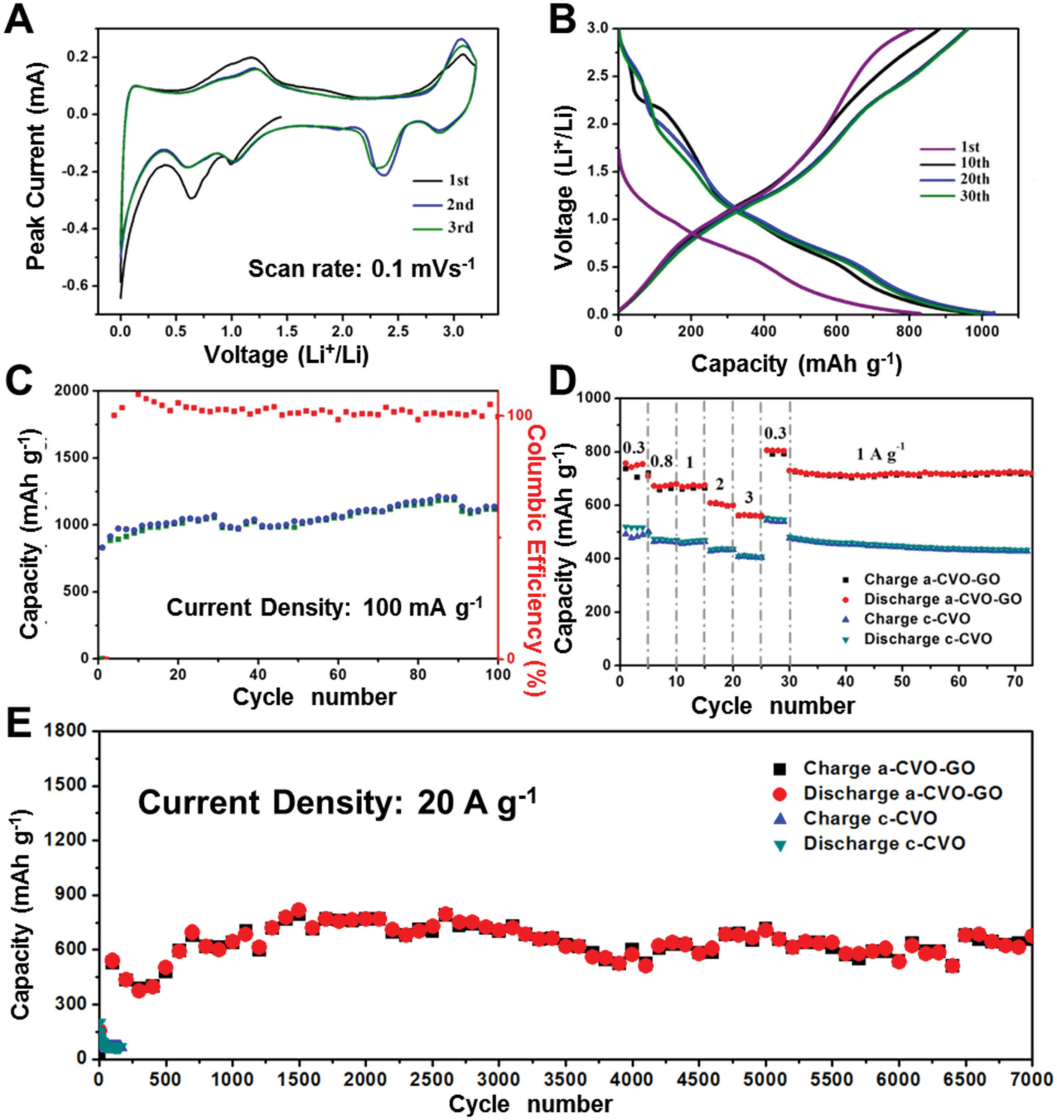
A) CV curves of a‐CVO‐GO at the scan rate of 0.1 mV s^−1^. The first three cycles are shown from the open circuit potential. B) Voltage profiles of a‐CVO‐GO for selected cycles at 100 mA g^−1^. C) Cycling performance of a‐CVO at 100 mA g^−1^. D) Rate performance of a‐CVO‐GO and c‐CVO. E) High‐rate long‐life cycling performance of a‐CVO and c‐CVO at 20 A g^−1^.

Figure 3E shows the cycling performance at an extremely high current density of 20 A g^−1^. The initial capacity of a‐CVO‐GO is 517 mA h g^−1^. Note that at a high current density of 20 A g^−1^, the capacity drops in the first 350 cycles, increases from 350 to 650 cycles, and then stabilizes, which may be attributed to high‐rate lithiation induced reactivation^44^ (Figure S10, Supporting Information). After 7000 cycles the capacity reaches 673 mA h g^−1^, demonstrating the excellent high‐rate capability and outstanding cyclability. The difference in capacity between a‐CVO‐GO and c‐CVO gets even greater at a high current density. The c‐CVO exhibits an initial capacity of 200 mA h g^−1^ at 20 A g^−1^, which quickly degrades to around 50 mA h g^−1^ after 150 cycles. Moreover, in order to prove that the results are reproducible, the cycling performance at 0.1 and 20 A g^−1^ and rate performance is evaluated again and the electrochemical performance is similar (Figure S11, Supporting Information). At an even higher current density of 50 A g^−1^ (Figure S12, Supporting Information), the a‐CVO‐GO delivers a capacity of 505 mA h g^−1^ after the first cycle, which is well above the theoretical capacity of commercialized graphite (372 mA h g^−1^). A capacity of 380.9 mA h g^−1^ can be retained after 100 cycles, corresponding to a capacity retention of 75%. However, fast capacity decay is observed after 140 cycles and this may be associated with the decomposition of electrolyte (Figure S13, Supporting Information). To determine the function of graphene oxide, the electrochemical performance of a‐CVO and a‐CVO‐GO is compared in Figure S14, Supporting Information. The a‐CVO shows inferior high‐rate cycling stability. This may attributed to the reason that the graphene oxide nanosheet is able to offer increased electron conductivity especially when the structure of the electrode is destroyed.

To explain the extremely high rate performance of a‐CVO‐GO, the CV curves at different scan rates from 1 to 10 mV s^−1^ were performed (**Figure** **4**A). The half‐cell was cycled at each scan rate for two cycles and the CV curves at each scan rate overlap quite well, indicating the excellent electrochemical reversibility. The lithium diffusion coefficient is determined by the Randles–Sevcik equation. The a‐CVO‐GO exhibits a high lithium diffusion rate of 6.78 × 10^−8^ cm^2^ s^−1^ (Figure 4B). As mentioned above, the a‐CVO‐GO exhibits a series of slopes without obvious plateaus in both lithiation and delithiation, which is similar to the electrode behavior of an electric double layer capacitor (EDLC). However, it is unusual for an EDLC electrode to deliver a high capacity of 1144.6 mA h g^−1^ at 100 mA g^−1^. Based on an estimated charge density of 30–50 μC cm^−2^,^45^ the a‐CVO‐GO with a surface area of 49.5 m^2^ g^−1^ may contribute to an EDLC capacity of 5.9–9.9 mA h g^−1^. To determine the charge storage mechanism, the cyclic voltammograms is obtained as a function of the scan rate (Figure 4C). The current values at two potentials (0.01 and 3.0 V) are plotted as a function of the square root of the scan rate (*v* ^1/2^) in Figure 4D. Both the lithiation and delithiation currents exhibit a linear relation with *v* ^1/2^. Clearly, the capacitor behavior (current ∝ *v*) is not observed, eloquently demonstrating that lithium ions/electrons are stored in the bulk of a‐CVO‐GO rather than at the surface at the potentials of 0.01 and 3 V.

**Figure 4:**
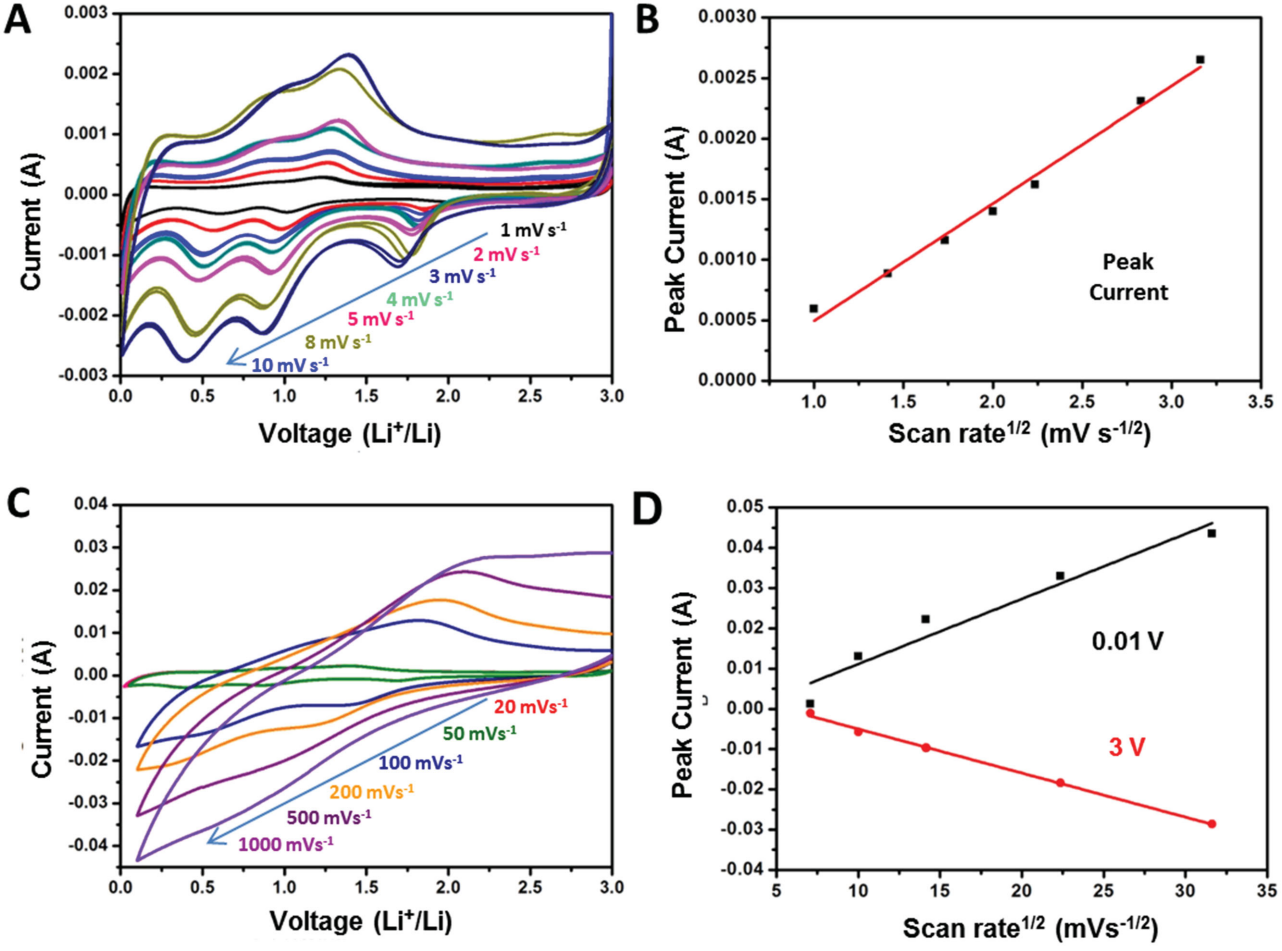
CV curves (A) at low scan rates ranging from 1 to 10 mV s^−1^. B) Randles–Sevcik plot obtained from the voltammetric data. CV curves C) at high scan rates ranging from 20 to 1000 mV s^−1^. Current versus square root of the scan rate D) at voltages of 0.01 and 3 V.

Next, we used the analysis proposed by Dunn and co‐workers^46^, ^47^ to further quantify the capacitive and diffusion limited contributions to the total capacity. In this method, the current at a particular voltage is regarded to be contributed from capacitive (*k*_1_) and diffusion (*k*_2_) controlled processes 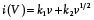



The amount of charge stored due to both diffusion and surface limited processes at different scan rates is presented in **Figure** **5**A. Generally, the capacitive charge storage does not vary significantly with the increase of sweeping rate; it fluctuates at around 440 mA h g^−1^. In contrast, with the increase of sweeping rate from 0.1 to 10 mV s^−1^, the diffusion controlled charge storage decreases from 831 to 71 mA h g^−1^. As a result, the contribution to the total capacity decreases from 65.3% to 16.0%. It is believed that the charge storage is mainly diffusion controlled in conventional lithium‐ion battery electrode materials. However, it is observed that the capacitive charge storage dominates the total capacity at sweeping rates above 0.5 mV s^−1^ in the a‐CVO‐GO. A typical separation of the capacitive and diffusion currents at a scan rate of 2 mV s^−1^ is shown in Figure 5B; the diffusion‐controlled charge storage is mainly occurred near the anodic/cathodic peaks, indicating that the diffusion controlled process is feasible at these regions. The high contribution of capacitive charge storage in our case might be attributed to the amorphous nature of a‐CVO, which provides more cation/anion vacancies, void spaces, cluster gaps or interstitial sites for lithium storage.^33^ More importantly, the dominant capacitive charge storage mechanism enables the high rate capability of a‐CVO‐GO.

**Figure 5:**
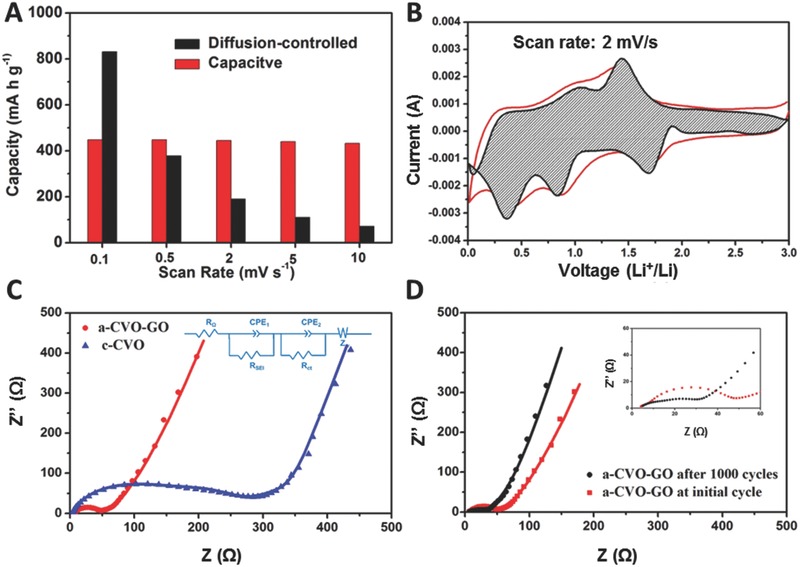
A) The capacity contribution at different scan rates (0.1, 0.5, 0.2, 2, 5, and 10 mV s^−1^). B) CV profile for a‐CVO‐GO at a sweeping rate of 2 mV s^−1^. The estimated capacitive current contribution is shown in the shaded region. C) Nyquist plots of a‐CVO‐GO and c‐CVO. D) Nyquist plots of a‐CVO‐GO before cycling and after 1000 cycles.

To provide further insights, the electrochemical impedance spectrum was measured. The a‐CVO‐GO shows a compressed semicircle in the high to medium frequency region and an inclined line (approximately 45°) in the low frequency range (Figure 5C). The compressed semicircle corresponds to the charge transfer resistance (*R*_ct_) of the electrode; while the inclined line stands for the Warburg impedance (*Z*_W_). The equivalent circuit model is provided in Figure 5C inset. In this circuit, *R*_Ω_ represents the Ohmic resistance of the electrode system, including the electrolyte and the cell components. *R*_SEI_ and *R*_ct_ represent the resistance related to solid electrolyte interface (SEI) and the charge transfer, respectively. CPE_1_, CPE_2_, and *Z*_w_ are the capacitance related to SEI, the double layer, and the Warburg impedance, respectively.^47^, ^48^ The charge transfer resistance of the a‐CVO‐GO is determined to be 50 Ω, much lower than that of the c‐CVO (284 Ω). Moreover, after 1000 cycles at 20 A g^−1^, the charge transfer resistance of a‐CVO‐GO becomes even lower (32 Ω, Figure 5D). This is due to the copper extraction during the first discharge process,^49^, ^50^, ^51^ which is demonstrated by the ex situ XRD pattern of the electrode that a peak at 42° is observed (Figure S15, Supporting Information). In this way, the in situ generated copper metal can enhance the electrical conductivity of the electrode.^36^, ^52^ Additionally, the slope in the low frequency range is increased, indicating the enhanced ion diffusion kinetics.

Based on the above analysis, the extraordinary high rate capability and excellent cycling performance is attributed to the aspects below. First, the amorphous nature of a‐CVO‐GO provides more active sites for lithium storage, leading to high capacitive charge storage.^33^ The dominant capacitive charge storage in turn endows fast charging and discharging. Second, the porous nanosheet building blocks of a‐CVO‐GO provide not only short lithium ion diffusion pathways but also high electrode‐electrolyte contact area. Third, the in situ extracted copper during the initial discharge process is able to enhance the electrical conductivity.

The amorphous copper vanadium oxide‐graphene oxide composite is synthesized through facile solution synthesis at room temperature via template‐engaged redox reaction followed by heat treatment in vacuum. The a‐CVO‐GO is able to deliver a capacity of 1145 mA h g^−1^ after 100 cycles at 100 mA g^−1^ and 672.5 mA h g^−1^ after 7000 cycles at 20 A g^−1^. Such outstanding electrochemical performance is contributed to the amorphous nature of a‐CVO‐GO and in situ generated copper during the initial discharge. The former enables the fast capacitive charge storage while the latter enhances the conductivity. Our strategy of designing amorphous ternary metal oxide with in situ formed metallic conducting network is demonstrated to be a promising method for developing novel high‐performance electrode materials for energy storage.

## Experimental Section

*Materials Synthesis: Preparation of the Cu_2_O Sphere*: The Cu_2_O sphere was prepared through a reported method.^51^ 1.0 g of PVP(K‐30) was dissolved in 50 mL of 0.01 m Cu(NO_3_)_2_ aqueous solution under rapid magnetic stirring. N_2_H_4_ solution (17 μL) was then introduced into the above solution. The orange colloidal solution was formed immediately. Under continual stirring for 40 min, Cu_2_O spheres were collected by centrifugation and washed with water and ethanol for several times.

*Preparation of a‐CVO‐GO and c‐CVO*: The graphene oxide was prepared by a modified Hummer's method. The fresh‐made Cu_2_O spheres were dispersed in 200 mL of distilled water. 50 mL of 0.02 m NH_4_VO_3_ aqueous solution was added to the Cu_2_O colloidal solution dropwise. Then 4 mL of the graphene oxide suspention (1 mg mL^‐1^) was added and ultrasoniccally dispersed. After stirring for 10 h, the color of the colloidal solution turned from orange to olive. Then the particles were collected by centrifugation and washed with distilled water and ethanol for three times. The precursor was desiccated at 70 °C in an oven. For the preparation of a‐CVO‐GO, the precursor was then transferred to the vacuum oven at 250 °C to remove the crystalline water. As a control experiment, the c‐CVO was synthesized by annealing the precursor at 450 °C in the air.

*Material Characterizations*: XRD patterns of the samples were collected with a D8 Advance X‐ray diffractometer with area detector, using Cu Kα radiation (λ = 1.5418 Å). The microstructures were observed by field‐emission scanning electron microscopy (JEOL‐7100F), transmission electron microscopy, and high‐resolution transmission electron microscopy (HRTEM) (JEM‐2100F). Brunauer Emmet‐Teller surface area was measured by using Tristar II 3020 instrument. Thermogravimetry is carried out on a STA449c/3/G (NETZSCH).

*Electrochemical Characterizations*: The electrochemical properties were characterized by assembing 2016‐type coin cells with lithium foil as the anode in a glovebox filled with pure argon. The cathode electrodes were composed of 60% active material, 30% acetylene black, and 10% sodium alginate binder. After coating onto copper foil, the cathode was cut into round slice with >>0.36 cm^2^ in area and >>0.1 mm in thickness. The mass loading of active material is 2.5–2.8 mg cm^−2^. A solution (1 m) of LiPF_6_ in EC/DMC (1:1 vol/vol) was used as the electrolyte. The cells were aged for 12 h before charge/discharge processes to ensure full penetration of the electrolyte into the electrodes. Galvanostatic charge/discharge measurements were performed by a multichannel battery testing system (LAND CT2001A). Cyclic voltammetry (0.01–3 V) was performed using an electrochemical workstation (CHI 660), electrochemical impedance spectroscopy (EIS) were tested with an Autolab Potentiostat Galvanostat (PGSTAT302N). All the measurements were carried out at room temperature. In order to perform the SEM images after cycling, the cells were disassembled and the cathode was dispersed in the alcohol for 72 h to remove the binder and the electrolyte.

## Supporting information

As a service to our authors and readers, this journal provides supporting information supplied by the authors. Such materials are peer reviewed and may be re‐organized for online delivery, but are not copy‐edited or typeset. Technical support issues arising from supporting information (other than missing files) should be addressed to the authors.

Supplementary
