# Supplementary material for: Graphene Oxide Wrapped Amorphous Copper Vanadium Oxide with Enhanced Capacitive Behavior for High‐Rate and Long‐Life Lithium‐Ion Battery Anodes
Source: Adv Sci (Weinh). 2015 Aug 7;2(12):1500154. doi: 10.1002/advs.201500154 (PMC5115307; doi:10.1002/advs.201500154)
Supplement: Supplementary file 1 — Supplementary [file ADVS-2-0b-s001.pdf]

Copyright WILEY-VCH Verlag GmbH & Co. KGaA, 69469 Weinheim, Germany, 2015.

ADVANCED SCIENCE
Open Access

Supporting Information

for *Adv. Sci.*, DOI: 10.1002/advs. 201500154

Graphene Oxide Wrapped Amorphous Copper Vanadium Oxide with Enhanced Capacitive Behavior for High-Rate and Long-Life Lithium-Ion Battery Anodes

*Kangning Zhao, Fengning Liu, Chaojiang Niu, Wangwang Xu, Yifan Dong, Lei Zhang, Shaomei Xie, Mengyu Yan, Qiulong Wei, Dongyuan Zhao, and Liqiang Mai**

Copyright WILEY-VCH Verlag GmbH & Co. KGaA, 69469 Weinheim, Germany, 2013.

Supporting Information

Graphene Oxide Wrapped Amorphous Copper Vanadium Oxide with Enhanced Capacitive Behavior for High-Rate and Long-Life Lithium-Ion Battery Anodes

*Kangning Zhao†, Fengning Liu†, Chaojiang Niu†, Wangwang Xu, Yifan Dong, Lei Zhang, Shaomei Xie, Mengyu Yan, Qiulong Wei, Dongyuan Zhao and Liqiang Mai**

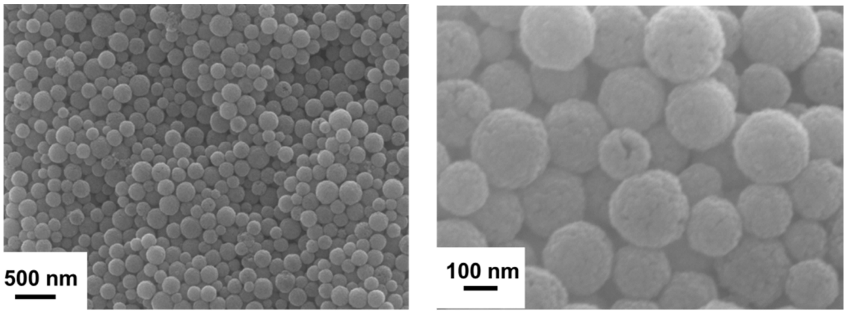

Figure S1. SEM images of Cu_2O spheres.

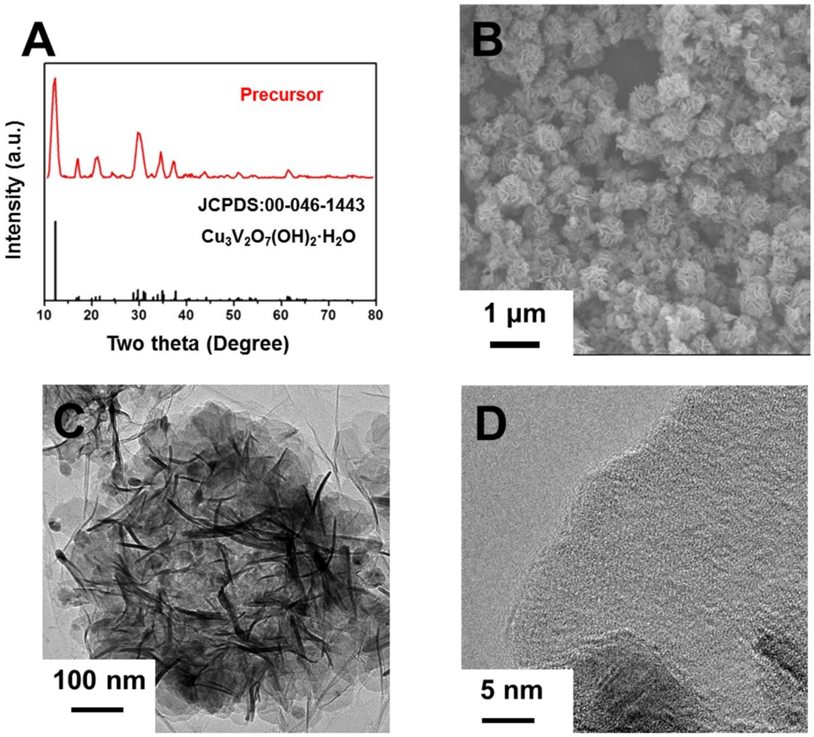

Figure S2. XRD pattern (A), SEM image (B), and TEM images (C, D) of the $Cu_3V_2O_7(OH)_2{\cdot}H_2O$ precursor.

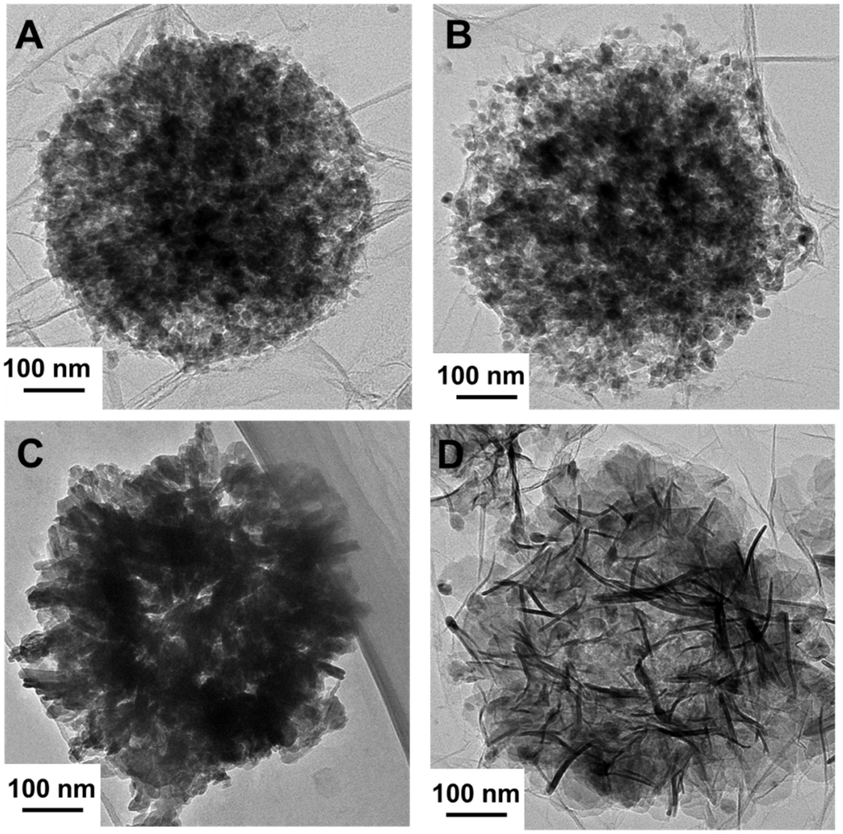

Figure S3. TEM images of the intermediates at different reaction time: 5 min (A), 30 min (B) , 1 h (C), 10 h (D).

Table S1. ICP results of a-CVO-GO and c-CVO.

	Mass concentration		Cu:V ratio
a-CVO-GO	Cu	46.54 %	1.57:1.00
	V	23.66 %	
c-CVO	Cu	45.39 %	1.59:1.00
	V	22.79 %	

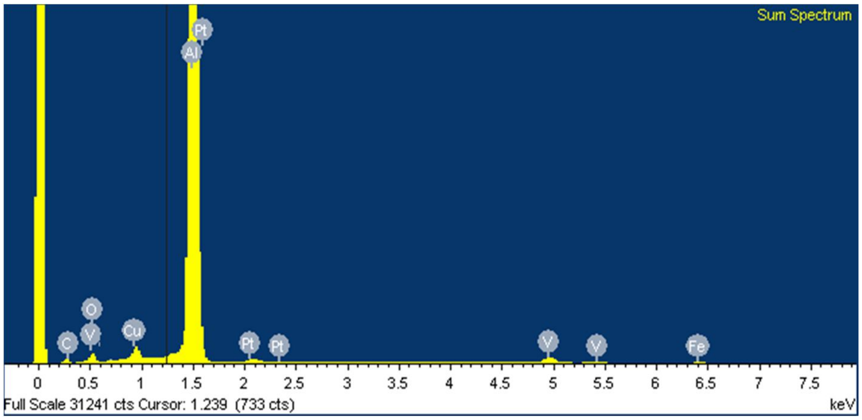

Figure S4. EDX measurements of a-CVO

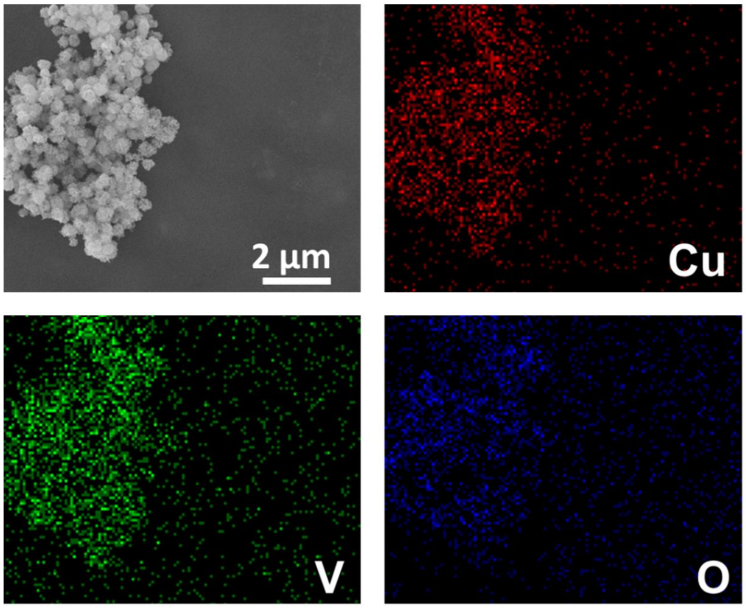

Figure S5. The elemental distribution of a-CVO (three elements, namely, Cu, V, O included).

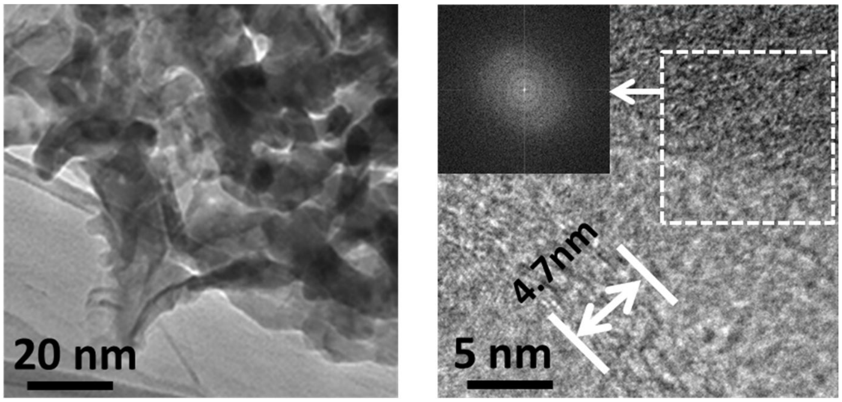

Figure S6. TEM images of a-CVO-GO (A, B). Inset of (B) is the Electron diffraction in the selected area.

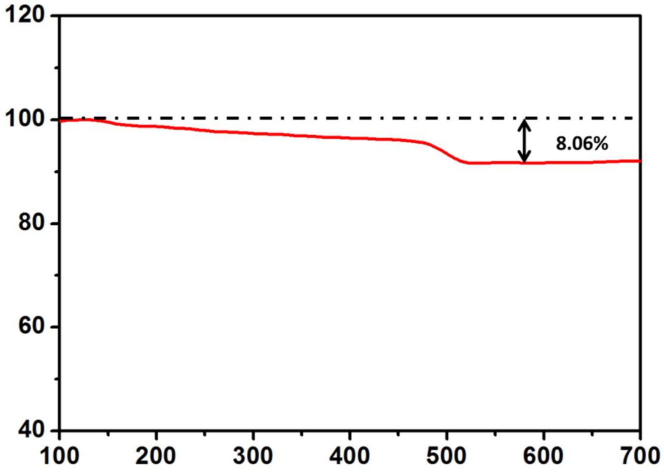

Figure S7. TG curve of a-CVO-GO in air.

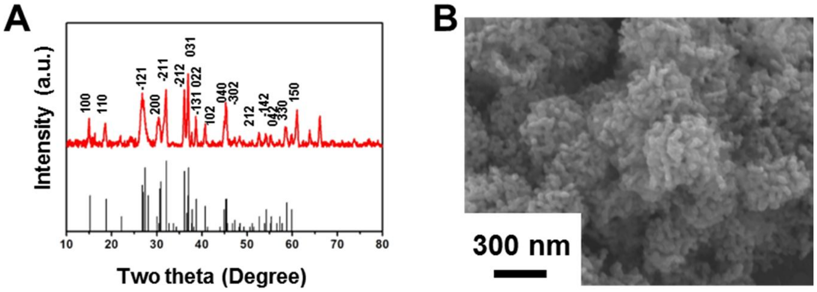

Figure S8. XRD pattern (A) and SEM image (B) of c-CVO.

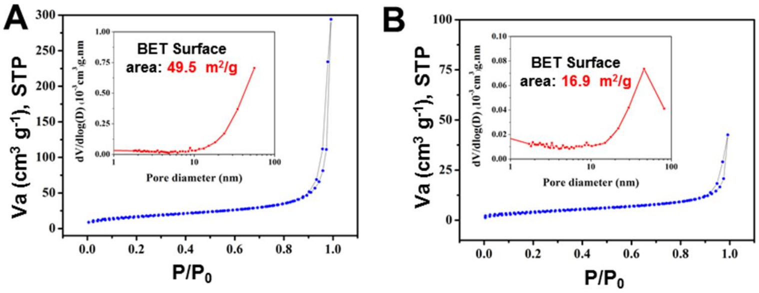

Figure S9. The nitrogen adsorption-desorption isotherms of a-CVO-GO (A) and c-CVO (B).

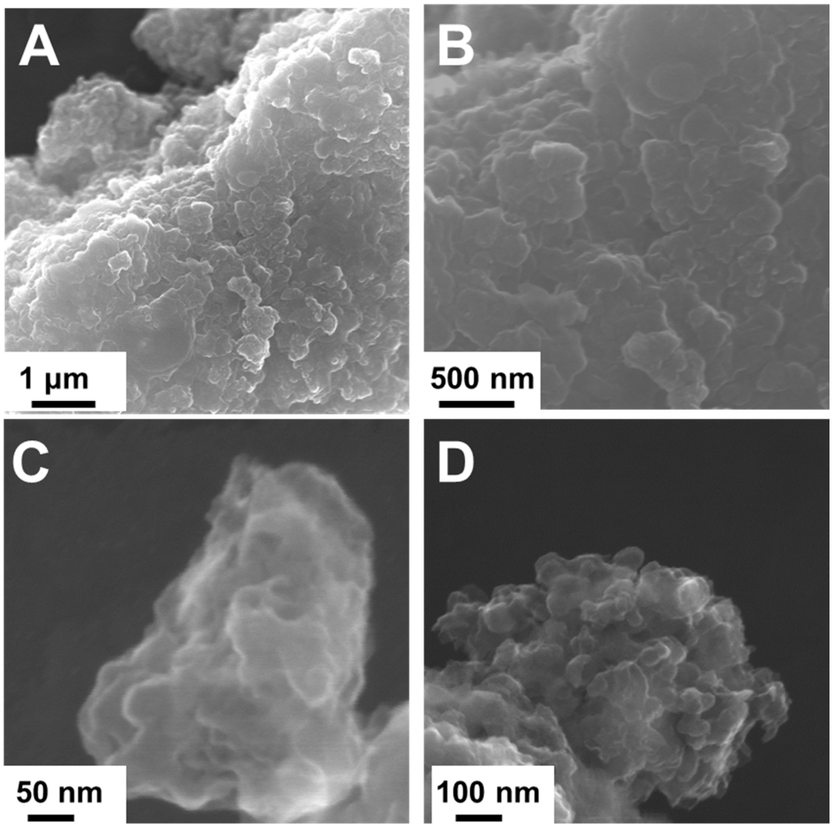

Figure S10. *Ex-situ* SEM image of a-CVO-GO after 100 cycles at current density of 100 mA g^{-1} (A, B) and 20 A g^{-1} (C, D).

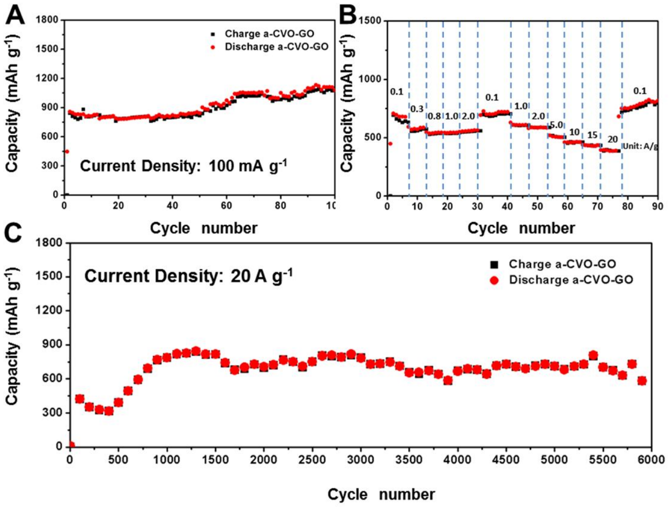

Figure S11. The reproduced electrochemcial performance: the cycling performance of at current density of 100 mA g^{-1} (A), the rate performance (B), the high-rate performance at rate of 20 A g^{-1} (C) of a-CVO-GO.

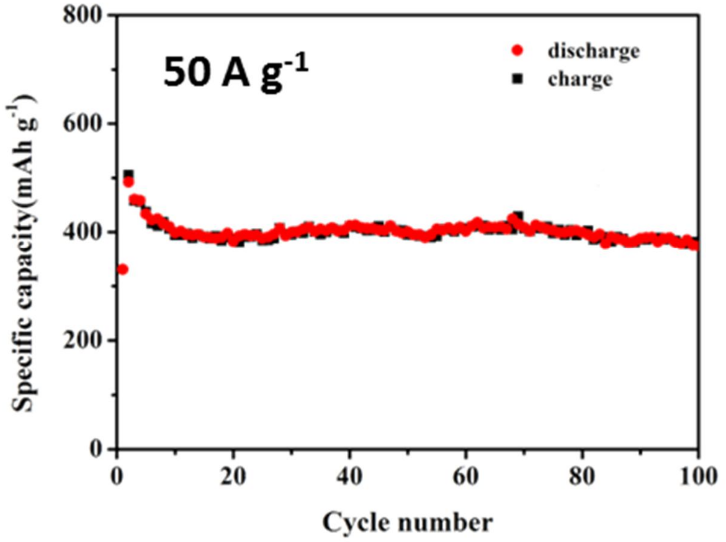

Figure S12. Cycling performance of a-CVO-GO at 50 A g^{-1}.

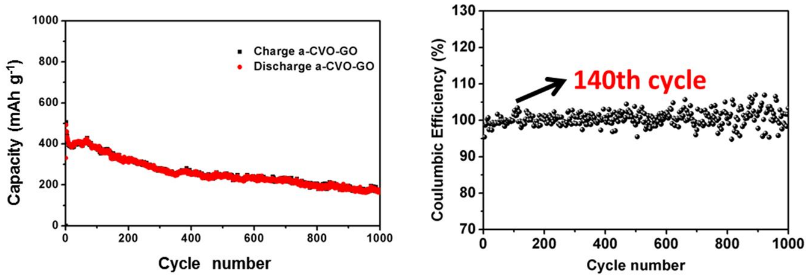

Figure S13. The cycling performance (A) of a-CVO-GO and the corresponding coulumbic efficiency (B) at current density of 50 A g^{-1}.

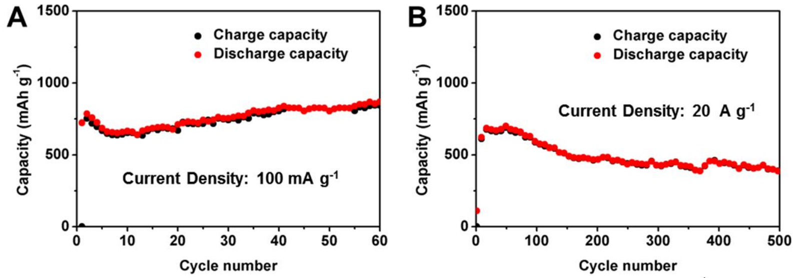

Figure S14. The cycling performance at current density of 100 mA g^{-1} (A) and 20 A g^{-1} (B).

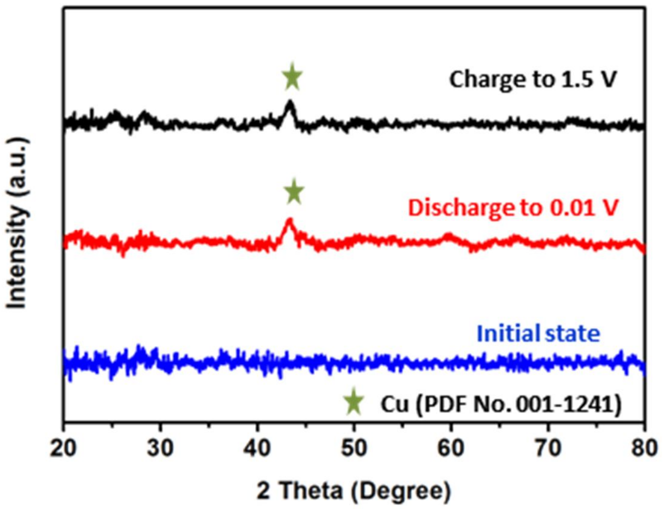

Figure S15. *Ex-situ* XRD patterns of the electrode at different voltage: initial state, discharged to 0.01 V, and charged to 1.5 V.
